# Willingness to Receive mHealth Services Among Patients with Diabetes on Chronic Follow-up in Public Hospitals in Eastern Ethiopia: Multicenter Mixed-Method Study

**DOI:** 10.2147/DMSO.S428210

**Published:** 2023-12-13

**Authors:** Dawit Firdisa, Admas Abera, Jerman Dereje, Fekede Asefa

**Affiliations:** 1 School of Public Health, College of Health and Medical Sciences, Haramaya University, Harar, Ethiopia; 2 Department of Psychiatry, College of Health and Medical Sciences, Haramaya University, Harar, Ethiopia; 3 Department of Pediatrics, College of Medicine, University of Tennessee Health Science Center (UTHSC) - Oak Ridge National Laboratory (ORNL) Center for Biomedical Informatics, Memphis, TN, 38103, USA

**Keywords:** willingness, mHealth, mobile phone, diabetes, mixed method, eastern Ethiopia

## Abstract

**Background:**

Management of diabetes requires a long-term care strategy, including support for adherence to a healthy lifestyle and treatment. Exploring the willingness of patients with diabetes to receive mHealth services is essential for designing efficient and effective services. This study aimedto determine willingness to receive mHealth services and associated factors, as well as explore the barriers to receive mHealth services among patients with diabetes.

**Methods:**

A multicenter mixed-method study was employed from September 1 to November 30, 2022. For the quantitative part, a total of 365 patients with diabetes receiving chronic follow-up at three public hospitals were enrolled. Data were gathered using structured questionnaires administered by interviewers, entered into Epi-data version 4.6, and analyzed using Stata version 17. A binary and multivariable logistic regression model was computed to identify the associated factors. For qualitative, eight key informants and seven in-depth interviews were conducted. After verbatim transcription and translation, the data were thematically analyzed using ATLAS.ti V. 7.5.

**Results:**

Overall, 77.3% had access to a mobile phone, and 74.5% of them were willing to receive mHealth services. Higher odds of willingness to receive mHealth services were reported among patients with an age below 35 years [AOR = 4.11 (1.15–14.71)], attended formal education [AOR = 2.63 (1.19–5.77)], without comorbidity [AOR = 3.6 (1.54–8.41)], <1-hour travel to reach a health facility [AOR = 3.57 (1.03–12.36)], answered unknown calls [AOR = 2.3 (1.04–5.13)], and were satisfied with health-care provider service [AOR = 2.44 (1.04–5.72)]. In the qualitative part, infrastructure, health facilities, socioeconomic factors, and patients’ behavioral factors were major identified barriers to receiving mHealth services.

**Conclusion:**

In this study, the willingness to receive mHealth services for those who have access to mobile phones increased. Additionally, the study highlighted common barriers to receiving mHealth services.

## Introduction

Diabetes mellitus (DM) is one of the main causes of morbidity and mortality, with over five million deaths estimated globally in 2017 due to diabetes.^2^ According to the International Diabetes Federation, 79% of patients with diabetes reside in low- and middle-income countries (LMICs), and the burden is expected to rise in the future.^3^ In 2019, there were 463 million estimated patients with diabetes globally, with the number anticipated to reach 700 million by 2045. **In** Africa, 24 million adults had diabetes in 2021, and Ethiopia was ranked fourth with regard to the number of patients with diabetes.^4^,^6^ A systematic review and meta-analysis in Ethiopia revealed that the prevalence of diabetes in the country was 6.5%.^3^ Currently, diabetes is a major area of focus for mHealth interventions, despite being underexplored in sub-Saharan Africa (SSA).^7^,^8^

mHealth is a medical and public health intervention assisted by mobile phones, patient monitoring devices, personal digital assistants, or other wireless technologies.^9^ mHealth services, including smartphone-based diabetes applications, played a significant role in diabetes prevention and follow-up intervention programs in high-income countries (HICs), where they are being utilized for bringing initiatives designed to promote a healthy diet, regular physical activity, prevent obesity, improve patient engagement in diabetes care, as well as reduce the number of care dropouts and glycemic control due to poor communication between the patient and the service providers.^10–14^

Poor communication between the patient and the health-care provider, the patients’ lack of knowledge regarding the disease condition and treatment, the complexity of the regimen, fear and experience of side effects, the cost of medication, and a lack of conviction about the necessity of treatment are all barriers to adherence to diabetes medication.^15–17^ Furthermore, adherence to diabetes medication and care remains a problem in LMICs.^18^,^19^ For instance, a study carried out in Ethiopia revealed that 25% of patients with diabetes properly followed their prescribed medications and dietary recommendations.^20^

These traditional barriers to accessing diabetes care can be overcome through the use of mobile phone devices in medical and public health practices.^21^ This approach offers advantages such as reducing travel and hospital waiting times, enhancing chronic disease prevention, patient care, and self-management, monitoring and provision of client-centered health information, lowering the cost of diabetes care, and improving the lives of underserved populations.^21–24^ Therefore, this study aimed to determine the willingness and associated factors among patients with diabetes for chronic follow-up in public hospitals in eastern Ethiopia.

## Methods and Materials

### Study Setting and Design

This study employed a multicenter institution-based sequential explanatory mixed-method approach in which a quantitative cross-sectional study was sequentially integrated into a qualitative phenomenological study. The study was conducted at three major public hospitals in eastern Ethiopia (Hiwot Fana Comprehensive Specialized University Hospital in Harar City, Dilchora Referral Hospital in Dire Dawa City, and Sheik Hassan Yabare Referral Hospital in Jigjiga City) from September 1–November 30, 2022. More than 2200 patients with diabetes were on follow-up during the study period in these three hospitals.

### Study Population and Sampling Approach

For quantitative part, the study populations were patients with diabetes (including both type I and type II) on chronic follow-up in selected public hospitals, whereas patients with diabetes and health-care providers (HCPs) in the selected hospitals were study participants for qualitative part. Patients with diabetes who have been diagnosed before 3 months of the data collection time, attending a diabetes follow-up clinic during the study period and willing to participate in the study were included in the study.

The sample size was determined using both a single and double population proportion formula. The maximum sample was achieved by using a double proportion formula by considering the assumptions: 95% confidence level, 80% power, and one-to-one ratio between case and control proportions: p1 = percentage of population who travel by car to come to a health facility (71.9%), p2 = percentage of population who travel by foot to come to a health facility (56.7%) based on a study conducted in northwest Ethiopia (23), and 10% of non-respondents. The final sample size was 365.

Systematic random sampling was employed to select study participants for the quantitative part. From each selected hospital, the number of participants included in the study was determined using proportional allocation based on their actual number of patients with diabetes. Respondents were selected while they were on their follow-up visits at the facility. On the first day of data collection, the first respondent was randomly selected from first six (k) respondents who came for the service followed by every other k^th^ respondent who was selected and interviewed until the required sample size was reached.

For the qualitative part, a purposeful sampling method was employed to select participants based on their diabetes information or experiences that members of the population were likely to possess. Seven in-depth interviews were conducted with patients with diabetes, and eight key informant interviews were held with health-care providers (nurse and physicians who provide services for patients with diabetes). Data collection was stopped when information saturation was reached, meaning that no additional data emerged during data collection.

### Data Collection and Quality Control Procedures

For the quantitative part, data were collected by 10 trained clinical nurses through face-to-face interviews using a structured questionnaire adapted from previous studies (23, 25) after being validated before use. Onsite supervision was performed by one data supervisor and the principal investigator. A pretest was done outside the study area on about 5% of the sample size. Moreover, data collectors were given two days of training with the aim of the study and the data collection tools. The completeness and quality of data collection were supervised daily, and feedback was provided to the data collectors.

For qualitative part, data were collected for 1 month in working hours each day. Participants were pre-identified and scheduled for interview. The data collector used an interview guide to facilitate interviews process. Guides were prepared in English, Amharic, Afan Oromo, and Af-Somali and were piloted in similar settings before being used for the study. Two trained data collectors experienced with qualitative data collection conducted in-depth interviews after 2-day training was given. Sony ICD PX470 sound recorder was used to record the participants’ responses. The interviews were conducted in a quiet and private room, which took about 20–40 min. The interviews were audio-recorded. Field notes were taken for all interviews to record any non-verbal observations. Upon completion of each in-depth interview, a trained language professional produced a complete transcript and translation of the data for data entry and analysis. Furthermore, transcriptions, translations, and coding of the data were reviewed with audio recordings to check for accuracy and authenticity. The rigor of the qualitative data was assured through thoughtful and deliberate planning, ongoing application of researcher reflexivity, and honest communication between the researcher and the audience regarding the study and its results.

### Data Analysis

For quantitative part, data were coded and entered using Epi Data V.4.6 and then exported to Stata V.17 for analysis. Frequency and cross-tabulations were used to describe the data. The associations between the characteristics of respondents and their willingness to receive mHealth services were analyzed using bivariable and multivariable logistic regression analyses. The backward selection method was used to select variables with a p-value of <0.25 in the bivariable logistic regression analyses. These variables were entered into a multivariable logistic regression to adjust for the effect of confounding. The model was fitted with the Hosmer and Lemeshow goodness-of-fit test of 0.6345, and a p-value of 0.05 was considered to be significant.

For qualitative part, the recorded Amharic and Afaan Oromo versions that were transcribed and translated back to English version. Field notes were expanded while on the field. Before themes and codes were identified, the researcher fully read and reread interview transcripts, independently identified possible themes and codes, and then pooled observations. Coding and analysis were done using Atlas.ti V.7.5 software. In general, inductive thematic analysis was used, in which themes were developed that represented the whole idea of categories.

## Results

### Quantitative Findings

#### Socio Demographic Characteristics of Participants

A total of 365 patients with diabetes have participated in this study. The age of the respondents ranges from 15 to 88 years, with a mean age of 48.3 years (standard deviation (SD) = 17.5). Overall, 51.5% of the respondents were females, 64.4% were married, and 62.7% attended primary or above educational level (Table 1).

**Table 1:** Socio-Demographic Characteristics of Patients with Diabetes on Chronic Follow-Up at Selected Public Hospitals, Eastern Ethiopia 2022

Variable		Frequency (N)	Percentage (%)
Sex	Male	177	48.5
	Female	188	51.5
Age	<35	99	27.1
	≥35	266	72.9
Residence	Urban	294	80.5
	Rural	71	19.5
Marital status	Single	82	22.5
	Married	235	64.4
	Separated	3	0.8
	Divorced	10	2.7
	Widowed	35	9.6
Religion	Orthodox	135	36.9
	Muslim	197	54
	Protestant	33	9.1
Education	No formal education	136	37.3
	Primary or above	229	62.7
Occupation	Government employed	71	19.5
	Merchant	53	14.5
	Farmer	53	14.5
	Housewife	88	24.1
	Other*	100	27.4
Income (USD)	<50	170	46.6
	≥50	195	53.4

**Notes**: *Other—retired, daily laborer, or private worker.

**Abbreviation**: USD, United States dollar.

#### Mobile Phone Access

More than three-quarters, 77.3% (95% CI: 72.6–81.3%), had access to a mobile phone, of which 37.9% were smart phones. Access to mobile phones was higher among urban residents (78.2%) as compared to rural residents (73.2%), among male (82.5%) compared female (72.3%) participants. Similarly, participants who earned $50 USD per month had higher access (87.2%) to mobile phone compared to who earned <$50 USD per month (65.9%) (Table 2).

**Table 2:** Mobile Phone Access Among Patients with Diabetes at Selected Public Hospitals, Eastern Ethiopia 2022

Variable		Access to Mobile Phone (n=365) n (%)	
		Yes	No
Sex	Male	146(82.5)	31(17.5)
	Female	136(72.3)	52(27.7)
Age	<30	49(68.1)	23(31.9)
	30-45	75(100)	0(0)
	>45	158(72.5)	60(27.5)
Residence	Urban	230(78.2)	64(21.8)
	Rural	52(73.2)	19(26.8)
Marital status	Single	61(74.4)	21(25.6)
	Married	188(80.0)	47(20.0)
	Separated	3(100)	0(0)
	Divorced	5(50.0)	5(50.0)
	Widowed	25(71.4)	10(28.6)
Religion	Orthodox	108(80.0)	27(20.0)
	Muslim	147(74.6)	50(25.4)
	Protestant	27(81.8)	6(18.2)
Education	Uneducated	60(55.1)	49(44.9)
	Educated	222(86.7)	34(13.3)
Occupation	Employed	69(97.2)	2(2.8)
	Merchant	50(94.3)	3(5.7)
	Farmer	39(73.6)	14(26.4)
	Housewife	54(61.4)	34(38.6)
	Others*	70(70.0)	30(30.0)
Income (USD)	<$ 50	112(65.9)	58(34.1)
	≥$50	170(87.2)	25(12.8)
Cohabitation status	Live alone	47(90.4)	5(9.6)
	With my spouse	129(79.1)	34(20.9)
	With family	100(71.4)	40(28.6)
	Others**	6(60.0)	4(40.0)
Having hypertension	Yes	93(67.4)	45(32.6)
	No	48(85.7)	8(14.3)
Having heart disease	Yes	50(76.9)	15(23.1)
	No	91(70.5)	38(29.5)
Type of diabetes	I	87(78.4)	24(21.6)
	II	195(76.8)	59(23.2)
Since diagnosis of diabetes (months)	<6	2(100)	0(0)
	6-12	19(79.2)	5(20.8)
	>12	261(77.0)	78(23.0)
Route of diabetes medication	Injection	112(78.9)	30(21.1)
	Oral hypoglycemic agent	170(76.2)	53(23.8)

**Notes**: *Others—retired, daily laborer, or private worker; others**—lives with relatives, lives with non-relatives.

#### Willingness to Receive mHealth Services

Among participants with access to mobile phone, willingness to receive mHealth services was 77.2% among females and 71.9% among males (Table 3). Additionally, 79.8% of participants who had access to television and 76.3% of those who had access to radio were willing to receive mHealth services (Table 4). From participants who reported being satisfied with their health-care providers, 78.2% expressed a willingness to receive mHealth services, compared to 59.6% among those who were not satisfied (Table 4). Furthermore, 75.9% participants who perceived benefit of mobile-based support for adherence were willing to receive mHealth service as compared to 69.3% among their counterparts (Table 4).

**Table 3:** Willingness to Receive mHealth Services Among Patients with Diabetes at Selected Public Hospitals, Eastern Ethiopia 2022

Variable		Willingness to Receive mHealth Services (n=282) n (%)	
		Yes	No
Sex	Male	105(71.9)	41(28.1)
	Female	105(77.2)	31(22.8)
Age	<30	46(93.9)	3(6.1)
	30-45	54(72.0)	21(28.0)
	>45	110(69.6)	48(30.4)
Residence	Urban	183(79.6)	47(20.4)
	Rural	27(51.9)	25(48.1)
Marital status	Single	53(86.9)	8(13.1)
	Married	139(73.9)	49(26.1)
	Separated	2(66.7)	1(33.3)
	Divorced	3(60.0)	2(40.0)
	Widowed	13(52.0)	12(48.0)
Religion	Orthodox	79(73.8)	28(26.2)
	Muslim	108(73.5)	39(26.5)
	Protestant	23(85.2)	4(14.8)
Education	Uneducated	32(53.3)	28(46.7)
	Educated	178(80.2)	44(19.8)
Occupation	Employed	60(87.0)	9(13.0)
	Merchant	39(78.0)	11(22.0)
	Farmer	23(59.0)	16(41.0)
	Housewife	38(70.4)	16(29.6)
	Others*	50(71.4)	20(28.6)
Income (USD)	<$ 50	77(68.8)	35(31.2)
	≥$50	133(78.2)	37(21.8)
Cohabitation status	Live alone	33(70.2)	14(29.8)
	With my spouse	96(74.4)	33(25.6)
	With family	77(77.0)	23(23.0)
	Others**	4(66.7)	2(33.3)
Having hypertension	Yes	61(65.6)	32(34.4)
	No	31(64.6)	17(35.4)
Having heart disease	Yes	32(64.0)	18(36.0)
	No	60(65.9)	31(34.1)
Type of diabetes	I	74(85.1)	13(14.9)
	II	136(69.7)	59(30.3)
Since diagnosis of diabetes (months)	<6	1(50)	1(50)
	6-12	14(73.7)	5(26.3)
	>12	195(74.7)	66(25.3)
Route of diabetes medication	Injection	91(81.3)	21(18.7)
	Oral hypoglycemic agent	119(70.0)	51(30)

**Notes**:*Others—retired, daily laborer, or private worker; others**—lives with relatives, lives with non-relatives.

**Table 4:** Willingness to Receive mHealth Services Stratified by Socioeconomic, Environmental And Behavioral Factors Among Patients with Diabetes at Selected Public Hospitals, Eastern Ethiopia 2022

Variable		Willingness to Receive mHealth Service (n=282) n (%)	
		Yes	No
Ownership of television	Yes	178(79.8)	45(20.2)
	No	32(54.2)	27(45.8)
Ownership of radio	Yes	87(76.3)	27(23.7)
	No	123(73.2)	45(26.8)
Availability of electricity	Yes	198(77.9)	56(22.1)
	No	12(42.9)	16(57.1)
Network availability	Yes	200(77.2)	59(22.8)
	No	10(43.5)	13(56.5)
How to travel to health facility	Walking	35(74.5)	12(25.5)
	By car	175(74.5)	60(25.5)
Time taken to reach the health facility	<1 hour	192(78.7)	52(21.3)
	≥1 hour	18(47.4)	20(52.6)
Had information about diabetes follow-up services	Yes	186(76.9)	56(23.1)
	No	24(60.0)	16(40.0)
Using reminder mechanisms	Yes	101(75.9)	32(24.1)
	No	109(73.2)	40(26.8)
Pill box	Yes	54(77.1)	16(22.9)
	No	47(74.6)	16(25.4)
Written schedule	Yes	40(76.9)	12(23.1)
	No	61(75.3)	20(24.7)
Watch alarm	Yes	2(100)	0(0)
	No	99(75.6)	32(24.4)
Satisfied with health care providers	Yes	176(78.2)	49(21.8)
	No	34(59.6)	23(40.4)
Missed appointments (in recent 1 year)	Yes	97(74.1)	34(25.9)
	No	113(74.8)	38(25.2)
Alcohol intake	Yes	24(72.7)	9(27.3)
	No	76(74.5)	26(25.5)
Chewing khat	Yes	71(73.2)	26(26.8)
	No	29(76.3)	9(23.7)
Smoking	Yes	9(56.3)	7(43.7)
	No	91(76.5)	28(23.5)

Table 5 shows willingness to receive mHealth services across patterns of mobile phone usage. Of participants who had access to mobile phones, 74.8% of smartphone owners had a willingness to receive mHealth services compared 74.3% participants without a smartphone. Similarly, participants who used their mobile phone for health communication were more willing (81.5%) to receive mHealth services than those who did not (71.6%). The willingness to receive mHealth services was higher among patients with diabetes who always held their mobile phone (75.8%) compared to those who held their mobile phone sometimes (70.4%). Patients who allowed others to use and access their mobile phones had a lower willingness to receive mHealth services than those who did not (71.7% vs 76.3%). Additionally, internet users via mobile phone showed a higher willingness to receive mHealth services compared to non-users (79% vs 70.5%).

**Table 5:** Willingness to Receive mHealth Services Stratified by Pattern of Mobile Phone Use Among Patients with Diabetes on Chronic Follow-Up in Public Hospitals, Eastern Ethiopia, 2022

Variable		Willingness to Receive mHealth Services (n=282) N (%)	
		Yes	No
Type of mobile phone	Smart	80(74.8)	27(25.2)
	Not smart	130(74.3)	45(25.7)
Ever used mobile phone for health communication	Yes	66(81.5)	15(18.5)
	No	144(71.6)	5728.4)
Have mobile phone within family other than you	Yes	193(75.4)	63(24.6)
	No	17(65.4)	9(34.6)
Ever used mobile phone as medication reminder	Yes	34(79.1)	9(20.9)
	No	176(73.6)	63(26.4)
Preferred mode of communication	Voice call	195(79.6)	50(20.4)
	Text message	15(40.5)	22(59.5)
How often do you have your mobile phone with you?	Always	172(75.4)	56(24.6)
	Sometimes	38(70.4)	16(29.6)
Mobile phone lost, damaged or stolen	Yes	105(77.2)	31(22.8)
	No	105(71.9)	41(29.1)
Have any other phone number	Yes	40(71.4)	16(29.6)
	No	170(75.2)	56(24.8)
Switch off mobile phone during daytime	Yes	80(76.2)	25(23.8)
	No	130(73.5)	47(26.5)
Times or places where mobile phone is not taken	Yes	111(76)	35(24)
	No	99(72.8)	37(27.2)
Time when do not answer unknown calls	Yes	128 (70)	55(30)
	No	82(82.8)	17(17.2)
Using password to lock phone	Yes	121(75.6)	39(24.4)
	No	89(73)	33(27)
Put mobile phone where others use and access	Yes	79(71.8)	31(21.2)
	No	131(76.2)	41(23.8)
Sharing mobile phone with another person in family	Yes	122(76.3)	38(23.7)
	No	88(72.1)	34(27.9)
Able to send text message using mobile	Yes	147(73.5)	53(26.5)
	No	63(76.8)	19(23.2)
Able to read text message using mobile	Yes	158(74.9)	53(25.1)
	No	52(73.2)	19(26.8)
Delete text message without reading it	Yes	54(78.3)	15(21.7)
	No	104(73.2)	38(26.8)
Likelihood of text message to be seen by others	Very likely	67(71.2)	27(28.8)
	Somewhat likely	83(82.2)	18(17.8)
	Somewhat unlikely	24(58.5)	17(41.5)
	Very unlikely	36(78.3)	10(21.7)
Use internet on the phone	Yes	105(79)	28(21)
	No	105(70.5)	44(29.5)
Use social media pages	Yes	99(76.2)	31923.8)
	No	111(73.0)	41(27.0)
Facebook	Yes	93(77.5)	27(22.5)
	No	6(60.0)	4(40.0)
Email	Yes	19(76.0)	6(24.0)
	No	80(76.2)	25(23.8)
Google	Yes	22(55)	18(45)
	No	77(85.6)	13(14.4)
Perceived benefit of mobile-based support for adherence	Yes	167(75.9)	53(24.1)
	No	43(69.3)	19(30.7)

**Figure 1:**
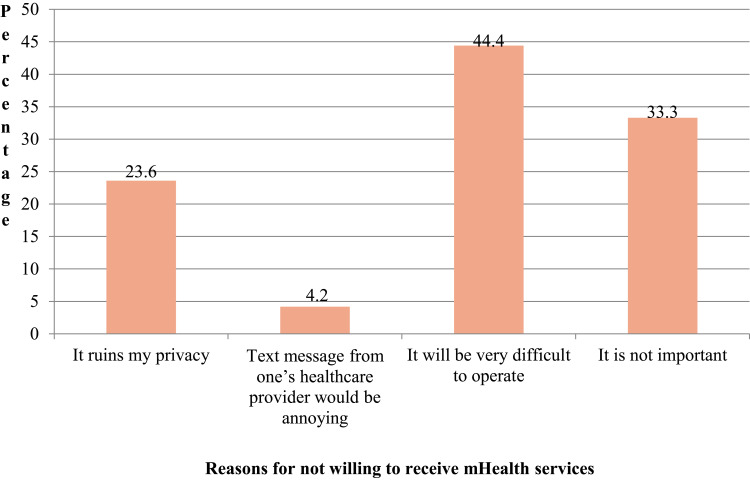
Reason for being unwilling to receive mHealth services among patients with diabetes on chronic follow up in public hospitals, eastern Ethiopia, 2022.

**Figure 2:**
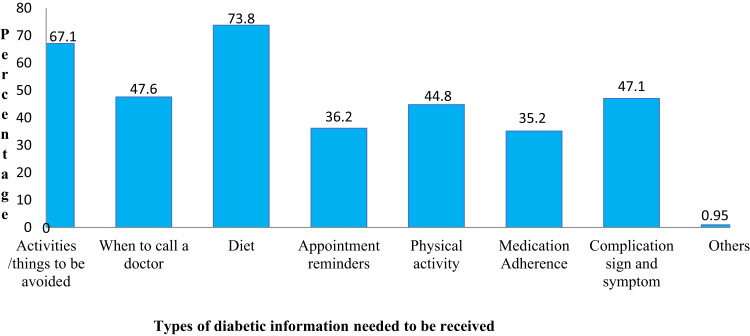
Diabetic information needed to be received by patients with diabetes on chronic follow up among those who are willing to receive mHealth services in public hospitals, eastern Ethiopia, 2022.

Among participants, 44.4% of who were unwilling to receive mHealth service reported difficulties in operating their phones and 33.3% perceived mHealth services were not important (Figure 1). Moreover, of individuals willing to receive mHealth services, 73.8% showed a willingness to receive dietary-related information; while 67.1% expressed interest in receiving guidance on things they should avoid (Figure 2).

#### Factors Associated with Willingness to Receive mHealth Services

Results of the bivariable analyses indicated that age, marital status, educational status, residence, occupation, average monthly income, types of diabetes, presence of comorbidity, route of medication, television ownership, time to reach the health facility, satisfaction with health-care providers’ service, having heard DM-related information, follow-up care services, open communication with health-care providers, availability of electricity, and availability of a telecommunication network were associated with willingness to receive mHealth service.

Moreover, previously using mobile phone for a health communication, changing mobile phone number in the last 12 months, not willing to answer calls from unknown number, using internet by their mobile phone, preferred mode of communications, locking mobile phone with passwords, access to mobile phone by others, current use of mobile phone for adherence, and availabilities of time or place for calls were significantly associated with willingness to receive mHealth services at a p value of <0.25. All of these associated factors were entered in the multivariable logistic regression analysis model to control for the effect of confounders.

Table 6 shows the result of the multivariable logistic regression model. The respondents with aged <35 years had 4.1 times more odds of willingness to use mHealth services than those 35 years of age and older [AOR=4.1 (95% CI: 1.2–14.7)]. Educated respondents had 2.6 times more odds of willingness to use mHealth services than uneducated respondents [AOR=2.6 (95% CI: 1.2–5.8)]. Patients with other comorbidities had 3.6 times more odds of willingness than those without comorbidities [AOR=3.6 (95% CI: 1.5–8.4)]. On the other hand, respondents who were satisfied with health-care providers had more odds to express their willingness to receive mHealth services than those who were not satisfied [AOR=2.4 (95% CI: 1.0–5.7)], while those who answer unknown calls were 2.3 times more willing as compared with those who do not answer unknown calls (2.3 (1.0–5.1)). Moreover, individuals who traveled for less than 1 hr had 3.6 times more odds to express willingness compared with those who traveled more than 1 hr (3.6 (95% CI:1.0–12.4)).

**Table 6:** Bivariable and Multivariable Analyses of Factors Associated with Willingness to Receive mHealth Services Among Patients with Diabetes on Chronic Follow-Up at Selected Public Hospitals, Eastern Ethiopia 2022

Variables		Crude OR (95%CI)	P-value	Adjusted OR (95%CI)	P-value
Age	<35	2.84(1.37-5.89)	0.005	4.11(1.15-14.71)*	0.030
	≥35	Ref	Ref	Ref	Ref
Marital status	Single	Ref	Ref	Ref	Ref
	Married	0. 43(0.19-0.96)	0.041	0.68(0.20-2.38)	0.547
	Others^#^	0.18(0.07-0.50)	0.001	0.40(0.10-1.71)	0.219
Diabetes types	I	Ref	Ref	Ref	Ref
	II	0. 41(0.21 -0.79)	0.008	2.1(0.76-5.76)	0.151
Television	Yes	3.34(1.82-6.13)	0.000	2.95(0.95-9.11)	0.061
	No	Ref	Ref	Ref	Ref
Education	No formal education	Ref	Ref	Ref	Ref
	Primary or above	5.62(3.15-10.04)	0.000	2.63(1.19-5.77)*	0.016
Occupation	Employed	Ref	Ref	Ref	Ref
	Merchant	0.53(0.20-1.40)	0.201	0.68 (0.20-2.34)	0.539
	Farmer	0.22(0.08-0.56)	0.002	0.72(0.16-3.32)	0.671
	Others^##^	0.37(0.16-0.82)	0.014	0.48(0.16-1.45)	0.190
Average monthly income (USD)	<50	Ref	Ref	Ref	Ref
	≥50	1.63(0.95-2.81)	0.075	2.1(0.98-4.31)	0.056
Is there comorbidity	Yes	Ref	Ref	Ref	Ref
	No	2.73(1.55-4.810)	0.000	3.6(1.54-8.41)*	0.003
Open communication with health professionals	Yes	Ref	Ref	Ref	Ref
	No	0.47(0.26-0.84)	0.011	0.58(0.25-1.34)	0.203
Tele network availability in house	Yes	Ref	Ref	Ref	Ref
	No	0.23(0.10-0.54)	0.001	0.27(0.07-1.04)	0.058
Travel time to reach the health facility	< 1 hour	4.1(2.02-8.32)	0.000	3.6(1.03-12.36)*	0.047
	≥1 hour	Ref	Ref	Ref	Ref
Times when not answering unknown calls	Yes	Ref	Ref	Ref	Ref
	No	2.07(1.13-3.82)	0.019	2.3(1.04-5.13)*	0.041
Probability of text message received on mobile phone seen by others	Very likely	Ref	Ref	Ref	Ref
	Somewhat likely	1.86(0.94-3.66)	0.073	2.3(0.97-5.45)	0.059
	Somewhat unlikely	0.57(0.27- 1.22)	0.149	0.38(0.13-1.11)	0.079
	Very unlikely	1.45(0.63 -3.33)	0.380	0.87(0.29-2.67)	0.814
Satisfied with health care providers	No	Ref	Ref	Ref	Ref
	Yes	2.43(1.31-4.50)	0.005	2.44(1.04-5.72)*	0.040

**Note**: *Significant at p-value <0.05, Others^#^-separated, divorced, and widowed, Others^##^-house wife, students, daily workers, and others.

### Qualitative Findings

#### Themes Identified During the Analysis

The analysis of 15 interviews (primary documents) resulted in 90 open codes being merged into 42 codes, then categorized under 11 subthemes (categories) from which four major themes (subcategories) were emerged. The key themes and sub-themes with identified barriers are shown in Table 7.

**1. Infrastructure Related Barriers**

**Table 7:** Themes and sub-themes emerged for qualitative study on barriers to receive mHealth services among patients with diabetes on chronic follow up in public hospitals, Eastern Ethiopia, 2022

Themes (Subcategories)	Subthemes(Categories)
Infrastructure related barriers	Lack of access to network
	Lack of access to electricity
Working environment/ health facility related barriers	Difficulty of applying equally to all patients
	Lack of sufficient number of health care professionals
	Potential lack of appropriate counseling from HCPs
Socioeconomic status related barriers	Not having mobile phone
	Economic status
Behavioral related barriers	Resistance to acceptance new services
	Low satisfaction toward mobile phone services
	Lack of awareness about mHealth services
	Lack of knowledge about mHealth services

**Lack of access to network**

This finding revealed that the network problem is one of the barriers towards willingness to receiving mHealth services among patients with diabetes on chronic follow-up.

A 42-year-old male patient stated his opinion as:

There might be a network problem especially for those who come from rural area where network can’t reach. One of the problems with a network is that it prevents you from meeting or communicating with many people, especially those who live in remote areas away from health facilities. (IDI-1)

**Lack of access to electricity**

The finding also depicted a lack of access to electricity as a barrier towards willingness to utilize mHealth services.

A 36-year-old female patient reported:

There are places in remote rural areas where electricity is not available. Because of this our mobile phone usually becomes off until we go to town to charge it, unless for those we charge by solar, if we own. This may be the reason why we don’t usually able to use such a new technology. (IDI-3)

**2. Socioeconomic Status Related Barriers**

**Ownership of mobile phone**

The results also demonstrated that willingness to receive mHealth services is hindered by not possessing a mobile phone.

A male medical doctor, age 26, stated:

First, it is questionable that all patients have access to a mobile phone. Not only mobile phone, but also a kind of mobile phone for using different applications like smartphone to utilize these services could also be another challenge. (KI-11).

**Economic status**

This study also indicated that low socioeconomic status is a barrier towards willingness to receive mHealth services.

A 29-year-old female nurse shared her experience as follows:

I think the inability to pay for this service could be one of the obstacles to receiving this service. The second is a lack of understanding about these mHealth services, so first giving information about mHealth services to patients is very important. (KI-15)

**3. Behavioral Related Barriers**

**Lack of awareness about mHealth services**

This study indicated that a lack of awareness about mHealth services is a barrier toward willingness to receive mHealth services among patients with diabetes on chronic follow-up.

A nurse who is 36 years old stated:

Patients with diabetes have no awareness about utilizing technology like mobile phone to receive health services. A lot of peoples even do not have any idea regarding mobile health services or they have no information or knowledge about mHealth services. (KI-14).

**Lack of knowledge about mHealth services**

This study also indicated that a lack of knowledge about mHealth services is a barrier toward willingness to receive mHealth services among patients with diabetes on chronic follow-up.

A 55-year-old male patient also expressed his idea as:

Lack of knowledge is one of the problems. Because, for example, if you ask me something about the disease, it is difficult for me to understand it because I don’t know the medical words that have no direct meaning to translate to our country’s language. Due to this reason, it could be difficult to apply this service to us. (IDI-9)

**Resistance to accept new services**

Again, the finding also indicated that a resistance to accept this new service is among the barriers toward willingness to receive mHealth services.

A 30-year-old male doctor shared his experience as follows:

What I think could be a problem is that when you introduce this new service, there could be a resistance to accept this service. For instance, I don’t use Telebir [an application used for digital money services] even though I have access and could possibly use it, but I am not interested in using this service. So, this could also be other challenges. (KI-12)

**Low satisfaction toward mobile phone services**

The study also showed that low satisfaction toward mobile phone services is a barrier toward willingness to receive mHealth services. A 65-year-old male patient described his experience as follows:

In terms of receiving health care services over mobile phone, it could be challenging for me to believe in being treated over the phone only without physical contact between patient and doctor. Because, as you know, I do not think it is worthwhile to be treated unless I have seen and reached out by health care professionals. (IDI-5).

**4. Working Environment Related Barriers**

**Difficulty of applying uniformly to all patients**

The study also identified the difficulty of applying uniformly to all patients as a barrier toward willingness to receive mHealth services. A 65-year-old male patient stated:

I think this will take a lot of time to implement it. Frist there could be a few numbers of patients who can read and use the application to use these services. Lack of knowledge to read and write the text messages and also there could be lack of understanding of the medical terms could be the major problems to implement these services. If we go down to those who come from rural area there is low proportion of people who can read and write. (KII-11).

**Potential lack of appropriate counseling from health professionals**

From the findings, we see that a lack of appropriate counseling from HCPs is one of the barriers toward willingness to receive mHealth services. A 42-year-old male patient stated his experience as follows:

There is no doctor who has paid attention to side effects. It is through our efforts that we know the follow-up situations. Most doctors, as far as I have seen, do not add or inform patients of the potential side effects of medications. They do not care about us. People hear about the side effects from outside as rumors. But it’s bad to do that. It is irrelevant to reduce medication amounts for fear of side effects because one is harming oneself. And these conditions exist. Of course, that is due to a lack of appropriate counseling from doctors. (IDI-1).

**Lack of sufficient number of health professionals**

The result also indicated that a lack of a sufficient number of HCPs is another barrier toward willingness to receiving mHealth services. A 30-year-old male medical doctor said that:

There is usually a lack of enough health care professionals to provide healthcare services. The proportion of healthcare professionals to patients in our country is very low; this could be the problem with the health system in our country. Even until this time, there are a lot of patients who are dying due to a lack of service because there are not enough healthcare professionals at the health facility. So, to implement this new service the number of health professionals should be increased. (KI-12)

## Discussion

In this study, access to mobile phone and willingness to receive mHealth services among those who have access to mobile phones becomes 77.3% and (74.5%), respectively. Age, educational status, time to reach the health facility, presence of comorbidity, satisfaction with the health-care provider, and willingness to answer unknown calls were significantly associated with willingness to receive mHealth services.

Access to mobile phone in this study was 77.3%, which is consistent with a study conducted on patients with diabetes in northwestern Ethiopia, 77.8%,^23^ on chronically ill patients in North Central Honduras (78.2%)^26^ and on ART patients (76.2%) in northwestern Ethiopia.^27^ The exponential growth in the global distribution of cell phones may be the cause of this similarity.

However, access to mobile phones in this study is higher than studies conducted on hypertension in Bangladesh (61.6%),^28^ This discrepancy may be due to differences in study settings because this study has been conducted in major towns of the region, which make the respondents more accessible to mobile phones. Also, the difference in socioeconomic status between diabetes and HIV/AIDS patients may be another reason.

Additionally, access to mobile phones in this study is lower than in studies conducted on HIV/AIDS patients in northwest Ethiopia (85.5%), patients with diabetes in southern Ethiopia (91.4%), chronic disease patients in India (81.5%), and patients with diabetes in Nigeria (97.3%).^21^,^29–31^ This discrepancy may result from regional variations in socioeconomic level, health literacy, and the acceptance of technology’s positive effects on human health.

In this study, 74.5% of patients with diabetes who had access to a mobile phone were willing to receive mHealth services, which is comparable to a study done on patients with diabetes in northwestern Ethiopia (70.5%)^23^ and Nigeria (72.6%).^21^ However, it is higher than studies conducted on patients with diabetes in southern part of Ethiopia (59.1%),^31^ chronic disease patients in India (60%),^30^ and patients with diabetes in northern Ethiopia (47.1%).^32^ The discrepancy could be due to differences in socio-economic and digital technology awareness among study participants. Willingness to receive mHealth services was lower than in a study conducted on chronic illnesses conducted in North Central Honduras (80%).^26^ This difference might be due to the difference in the ICT development index and socioeconomic status across countries.

Having attended a formal education was positively associated with the willingness to receive mHealth services. This is a robust finding from numerous studies on various health programs, including ART patients, child immunization, and pregnant women receiving antenatal and prenatal care, which were carried out in the majority of LMICs.^21^,^23^,^25–27^,^33^ The observed association may be due to the likelihood that increased educational status will boost awareness of managing diabetes and provide greater access to a cell phone and mobile network.

Age is also another positively associated factor with willingness to receive mHealth services. This finding is in line with previous studies.^25^,^27^,^33–35^ The findings suggest that younger age groups are the most suitable for adopting mHealth services because they are more exposed to technology than older people.

Those who answer unknown calls are also positively associated with willingness to receive mHealth services. This finding is in line with study done in Ethiopia among ART patients.^27^ This similarity may be due to those who answer unknown calls and are mainly interested in using mHealth services.

Time taken to reach a health facility is also another significant associated factor for willingness to receive mHealth services. Those who traveled less than 1 hr were more likely to be willing compared with those who travel greater than 1 hr. This finding is in line with studies done in Ethiopia among ART patients.^27^ However, this contradicts a study conducted on patients with diabetes in northwestern Ethiopia.^23^ This disparity might be due to differences in study settings and characteristics of study participants.

Furthermore, those who are satisfied with health-care providers were more likely to be willing than those who did not satisfied. This result is consistent with studies conducted on patients with diabetes in northwestern Ethiopia.^23^ The similarity could be due to those who were satisfied with HCPs may have strong confidence on them that may increase their willingness.^36^ Also, those who have comorbidities were more likely to be willing than those who have no comorbidities. This is because comorbidity might have profound effects on patients’ ability to manage their self-care and pose significant barriers to lifestyle changes that make them less willing.

Qualitative study mainly focused on identification of barriers to receive mHealth services among patients with diabetes on chronic follow-up in public hospitals in eastern Ethiopia. The findings of this study identified infrastructure, health facilities, socioeconomic factors, and patients’ behavioral related barriers to receiving mHealth services.

This finding is consistent with a study conducted to explore facilitators of and barriers to mHealth adoption in older adults with heart failure, which shows barriers identified are lack of knowledge regarding how to use mHealth, cost of technology, and limited or fixed income.^37^ Our finding is also in line with the study conducted to assess barriers to and facilitators of the use of mobile health apps from a security perspective, indicating that the cost of apps and lack of security features in mHealth apps were barriers to adoption of mHealth apps.^38^

## Limitations of the Study

This study is not without limitations. Since the study is institution-based, only respondents who came for follow-up services during data collection time were interviewed. Moreover, the study was done at hospitals in a major town administration, which would have overstated the accessibility of patients with diabetes to mobile phones and their willingness to receive mHealth services, which may affect the generalizability of the findings. The survey was also interviewer-administered, there may be a risk of social desirability bias that would have made more participants respond as willing. The willingness was also assessed only for those who already have a mobile phone, so the generalizability is restricted to only those who access mobile phones.

## Conclusion

Our findings identify a high willingness to receive mHealth services among those who have access to mobile phones. Age, educational status, time to reach the health facility, presence of comorbidity, satisfaction with the health-care provider, and willingness to answer unknown calls are significantly associated with willingness to receive mHealth services.

Additionally, qualitative findings identified infrastructure, health facilities, socioeconomic factors, and patients’ behavioral factors as major barriers to receiving mHealth services. Therefore, implementing mHealth services for self-care, adherence support, and behavioral counseling and to improve patients’ knowledge might change the livelihood of patients with diabetes.

## Abbreviations

BP, High Blood Pressure; DM, Diabetes mellitus; HCPs, Health-care providers; HICs, High-income countries; HTN, Hypertension; ICT, Information and Communication Technology; IDF, International diabetes federation; IDIs, In-depth Interviews; IT, Information Technology; KI, Key informants; LMICs, Low- and middle-income countries; mHealth, Mobile Health; MMR, Mixed-method research; NCDs, Non-Communicable Diseases; SMS, Short/text Message Service; SSA, sub-Saharan Africa; WHO, World Health Organization.

## Data Sharing Statement

The datasets used during the current study are available from the corresponding author upon reasonable request.
